# Digital health and wearable devices for retinal disease monitoring

**DOI:** 10.1007/s00417-024-06634-3

**Published:** 2024-09-19

**Authors:** Malena Daich Varela, Alejandro Sanders Villa, Nikolas Pontikos, Michael D. Crossland, Michel Michaelides

**Affiliations:** 1 Moorfields Eye Hospital, London, UK; 2 UCL Institute of Ophthalmology, University College London, 11-43 Bath Street, London, EC1V 9EL UK; 3 Facultad de Enfermería y Obstetricia, Universidad Nacional Autónoma de México, Mexico City, México; 4 Primero Salud, Mexico City, México

**Keywords:** Retina, Digital health, Wearables, Self-monitoring, Clinical research

## Abstract

Digital health is wielding a growing influence across all areas of healthcare, encompassing various facets such as telemedicine, artificial intelligence (AI), and electronic healthcare records. In Ophthalmology, digital health innovations can be broadly divided into four categories: (i) self-monitoring home devices and apps, (ii) virtual and augmented reality visual aids, (iii) AI software, and (iv) wearables**. **Wearable devices can work in the background, collecting large amounts of objective data while we do our day-to-day activities, which may be ecologically more valid and meaningful to patients than that acquired in traditional hospital settings. They can be a watch, wristband, piece of clothing, glasses, cane, smartphone in our pocket, earphones, or any other device with a sensor that we carry with us. Focusing on retinal diseases, a key challenge in developing novel therapeutics has been to prove a meaningful benefit in patients’ lives and the creation of objective patient-centred endpoints in clinical trials. In this review, we will discuss wearable devices collecting different aspects of visual behaviour, visual field, central vision, and functional vision, as well as their potential implementation as outcome measures in research/clinical trial settings. The healthcare landscape is facing a paradigm shift. Clinicians have a key role of collaborating with the development and fine-tuning of digital health innovations, as well as identifying opportunities where they can be leveraged to enhance our understanding of retinal diseases and improve patient outcomes.

## Key message

***What is known***

- Digital health is changing the healthcare landscape, facilitating data acquisition, analysis and interpretation.
- Wearable devices in Ophthalmology can collect large amounts of objective data about the visual experience while we do our day-to-day activities.

***What is new***

- We provide an overview of new wearable technology that can become relevant in retinal disease clinical and research monitoring.
- Although many challenges are to be overcome, digital health and wearable devices in Ophthalmology are an interesting approach to improve our understanding of diseases, patient care and research.

## Introduction

Digital health is wielding a growing influence across all areas of healthcare, with an ever-expanding role in improving the accessibility and demographics of healthcare systems. It encompasses various facets including telemedicine, remote monitoring, artificial intelligence (AI), data analytics, electronic healthcare records, mobile health applications (apps), virtual and augmented reality (VR and AR) instruments, and wearable devices [1]. The combination of digital health with cyber physical systems and the Internet of Things (IoT; known in this context as the Internet of Medical Things) [2] spawned a network of electronic devices equipped with software, sensors, and network connectivity, enabling real-time big data collection and cloud storage that can be accessed remotely [3–5]. The subsequent introduction of AI and advances in hardware facilitated data analysis and interpretation, having a significant impact in all life and medical sciences [6]. These systems permit patient characterisation, staging, monitoring, and triaging in a fast and efficient manner, potentially accelerating research, diagnosis, and treatment [7], with some examples including: wearable continuous electrocardiogram monitoring [8], home 24-h urine analysis [9], detector of spectacles use compliance [10], and sweat-glucose skin sensors for constant glucose monitoring [11].

In Ophthalmology, digital health innovations can be broadly divided into four categories: (i) self-monitoring home devices and apps, (ii) VR and AR visual aids, (iii) standalone AI software, and (iv) wearables [12]. Remote monitoring includes assessment of intraocular pressure with self-tonometers [13], macular thickness with portable OCTs [14], and visual acuity (VA) [15] with mobile apps [16]. VR and AR low vision aids are able to afford visual field expansion, improved night vision and sharper VA in individuals with low vision through different types of headsets [17]. AI-mediated software is continuously growing, enabling bulk analysis of retinal images and presumptive diagnoses [18, 19]. Wearable devices have the capability of working in the background, collecting data while we do our day-to-day activities, sometimes also aiding with difficult tasks, and notifying the patient’s care network of any issues [20]. They can be a watch, wristband, piece of clothing, glasses, cane, smart phone in our pocket, earphones, or any other device with a sensor that we carry with us [21]. Combinations of the above categories (such as a wearable device with AR or with an AI analytics software) have the capacity to take it a step further, collecting data while improving the visual experience of the patients, or storing and analysing data in real-time, drawing patterns and associations, and connecting with the individual’s healthcare records.

Focusing on retinal diseases, a key challenge in the development of novel therapeutics for rare diseases (such as inherited retinal dystrophies -IRD-) has been to prove a meaningful benefit in patients’ lives and the creation of objective patient-centred endpoints in clinical trials [22]. Wearable devices are able to acquire large amounts of objective data which may be more ecologically valid and meaningful to patients [23] than that acquired in traditional hospital settings; at a minimum, complementing one another. Although most wearable devices are in a trial/prototype phase and require standardisation and further validity testing, [13] regulatory agencies such as the US Food and Drug Administration (FDA) and the European Medicines Agency (EMA) have encouraged the inclusion of patient monitoring devices as exploratory endpoints in clinical trials [24]. Furthermore, vulnerable members of society, such as individuals with visual impairment, may benefit greatly from AI-mediated sensors embedded in their houses, preventing health issues and alerting their network of any possible accident [25].

**Fig. 1:**
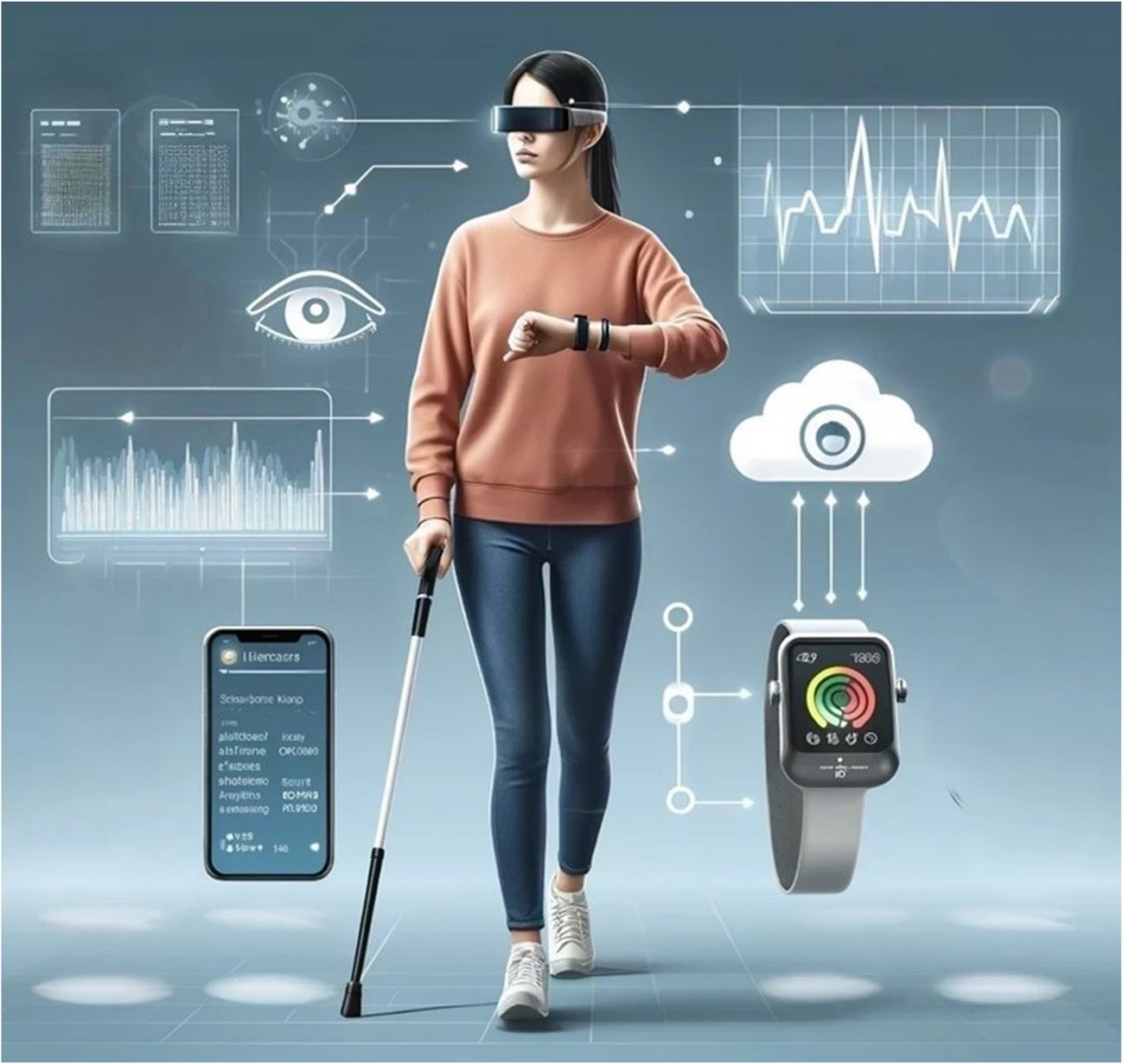
Visually impaired woman walking and using multiple wearable devices that interact with each other, collecting different types of data about the visual experience, having this stored and analysed in real time in the cloud. Image generated by artificial intelligence, OpenAI DALL-E, ChatGPT (2024)

In this review, different wearable devices relevant for retinal disease monitoring will be discussed, as well as their potential implementation as outcome measures in research/clinical trial settings (Table 1, Fig. 1).

**Table 1:** Current available wearable devices relevant for retinal disease monitoring. NA: Not applicable/available

Name	Area of vision to monitor	Type of wearable	Wearable role	Sample size	Conclusion	Author (year)
Clouclip (Glasson Technology Co. Ltd., Hangzhou, China)	Visual behaviour	Spectacle-attached	Measures working distance and eye-level light intensity	78 fifth-grade students from urban and rural schools	The device was able to detect substantial differences in light exposure and near-work metrics between the two regions	Wen et al. (2019) [26]
Vivior AG (Zurich, Switzerland)	Visual behaviour	Spectacle-attached	Captures working distance, vision duration and breaks, level of illumination, and head translational and rotational movements on the three axes	129 patients	87% of patients felt comfortable using the wearable device and 91% found it easy to attach to the magnetic clip	Pajic et al. (2019) [27]
JINS MEME (Japan)	Visual behaviour	Sensors located on glasses nose pads	Captures eye movements and blinking by electrooculography	5 healthy participants	Further verification and calibration are needed, with hardware possibly being too sensitive	Trzepacz et al. (2019) [28]
Actiwatch (Philips Respironics, USA)	Visual behaviour	Wristwatch	Measures light exposure and activity levels	NA	NA	[29]
Daysimeter-D (Lighting Research Center, USA)	Visual behaviour	Spectacles/shirt collar/hat-attached	Measures light exposure and activity levels	NA	NA	[29]
AMS AS7264A (Italy)	Visual behaviour	Forehead-mounted	Quantifies the time spent in front of a digital screen and connects with a smartphone through Bluetooth to store and analyse the data	10 healthy participants	The features are captured appropriately and with high accuracy	Martire et al. (2018) [30]
Tiger (Taiwan)	Visual behaviour	Spectacles with multiple sensors	Screen viewing (colour) and eye-resting detectors (head movement and viewing distance sensor)	10 healthy participants	Accurate detection of screen viewing events, with positive perception of usefulness and acceptance	Min et al. (2019) [31]
AR DSpecs (Bascom Palmer Eye Institute, USA)	Visual field (VF)	Spectacles with integrated AR technology	While calibrating, the glasses identify the size and location of scotomas and fit video images of the unseen field into the remaining VF. It also carries an eye tracking system that can monitor gaze and fixation.	21 patients with bilateral peripheral VF defects	AR DSpecs may improve walking maneuverability by enhancing object detection	Sayed et al. (2020) [32]
NA	Central vision	Head-mounted display with VR/AR and mixed reality	To identify, characterize, and monitor a scotomatous monocular region, and modify it through binocular suppression	18 healthy individuals with simulated scotoma	This technology showed digital suppression of monocular central visual distortions in early validation studies	Ong et al. (2022) [33]
Orcam MyEye (Jerusalem, Israel)	Central vision	Spectacle-attached	AI-powered real-time print-to-speech device integrating video and audio processing for reading, face recognition, identifying currency, and colours	100 visually impaired individuals	Statistically significant increase in patient’s ability of performing tasks. Most patients were pleased to use the device and didn’t experience problems	Amore et al. (2023) [34]
Wearable Virtual Cane Network (University of Georgia, USA)	Navigation	Four sensors in the waist, wrists and ankle and a micro-vibration motor	Hands-free aid that acquires whole-body navigation details and speed	7 blindfolded healthy participants	The results showed that the walking speed for an obstacle course was increased by 23% on average when subjects used the Wearable Virtual Cane Network rather than a white cane	Gao et al. (2015) [35]
U-HAR (University of Tübingen, Germany)	Visual behaviour	Spectacles with multiple sensors	A combination of a commercial eye-tracker, an inertial measurement unit and a convolutional network to capture eye and head movement features of 7 activities	20 healthy participants	The model achieved 86.59% accuracy detecting contextual information	Meyer et al. (2022) [36]
JINS MEME (Japan)	Visual behaviour	Sensors located on glasses nose pads	It allows offline classification of participants’ activities based on the collected data	12 healthy adults	Equal or better results with more diverse activities than other approaches involving multiple wearable devices, indicating that JINS MEME is able to recognize activities of daily living	Diaz et al. (2018) [37]
UCA-EHAR (Université Côte d’Azur, France)	Visual behaviour	Spectacles with multiple sensors	It allows offline classification of participants’ activities based on the collected data	20 healthy adults	Using a small neural network, the device can run human activity recognition for up to 24 hs	Novac et al. (2022) [38]
LV-3 (Harvard Medical School, USA)	Navigation	See-through spectacle-mounted device	Incorporates visual field expansion through minification and contour augmented view for improved night mobility	6 patients with night blindness	With improved camera sensitivity, patients might be able to have improved outdoor night-time mobility	Bowers et al. (2004) [39]
NA	Navigation	See-through spectacles	Incorporates visual field expansion through minification and increased brightness taken by a high-sensitivity camera for improved night-time mobility	28 patients with retinitis pigmentosa	Binocular visual acuity in the dark was significantly improved. In the walking test, the number of errors decreased greatly and the travel time was significantly shortened	Ikeda et al. (2019) [40]
NA	Head positioning	Soft headgear, composed of a headband with a vertical strip crossing the top of the head	Head positioning sensor which provides real-time audiovisual feedback on the accuracy of positioning	8 healthy volunteers	Improved positioning compliance in half of the cohort. This device could be used for postoperative positioning after retinal detachment repair with intraocular gas.	Brodie et al. (2017) [41]

## Visual behaviour

Visual behaviour can be defined as how people use their vision, and it generally refers to gaze direction, gaze movements, head movements, visual interaction, and the distance and level of illumination at which tasks are performed [42, 43]. These metrics provide valuable information about a person’s overall visual experience and have been largely studied in myopia [44–46] and communication/marketing studies [43].

To capture some visual behaviour components, Glasson Technology Co. Ltd. (Hangzhou, China) developed Clouclip, a spectacle-attached wearable device that measures working distance and eye-level light intensity, being able to track and quantify the risks of myopia development [26]. Combined with AI, it may also predict the development and progression of myopia [47], and through a vibrating feature, alert the user of unhealthy visual behaviours (near-work distance < 30 cm and > 5 s, or < 60 cm for > 45 min) [48].

Another device is the Visual Behaviour Monitor (Vivior AG, Zurich, Switzerland) which can also be fixed on spectacles, capturing the distance at which patients’ visual activities are performed, vision duration and breaks, the level of illumination, and head translational and rotational movements in three axes [27]. Another approach by Trzepacz et al. is to capture eye movements and blinking by electrooculography sensors located on the nose pads of JINS MEME ES glasses, which output the data to a smartphone [28].

Further devices are designed to measure light exposure and activity levels, such as the Actiwatch (Philips Respironics, USA), which works as a wristwatch, and the Daysimeter-D (Lighting Research Center, USA), that can be attached to spectacles or clipped on a shirt collar or hat [29]. Martire et al. introduced a forehead-mounted tri-stimulus colour light sensor (AMS AS7264A) which quantifies the time spent in front of a digital screen and connects with a smartphone through Bluetooth to store and analyse the data [30]. A similar device is Tiger, smart glasses equipped with screen viewing (colour) detector, eye-resting detector (head movement and viewing distance sensor), and real-time feedback (vibration) manager [31].

Environmental factors are becoming useful for preventive medicine, and are currently assessed by patient reports through questionnaires, possibly having recall bias [46]. In a study comparing wearable-derived data versus questionnaire data, participants tended to overestimate time spent outdoors and intermediate viewing [49]. Data regarding the light at which patients navigate or do their activities is relevant while characterising retinal diseases such as achromatopsia or retinitis pigmentosa (RP), being associated with photoreceptors function and disease severity [50, 51].Wearable devices are able to provide objective data, representative of real-life patients’ settings, and detect changes over time, valuable for baseline and longitudinal assessments.

## Visual field

Patients with IRD that primarily affect rod photoreceptors (e.g., RP) have peripheral field constriction as one of the cardinal symptoms. Different headsets exist in the market for mobile visual field (VF) assessments, with good correlation with traditional perimeters in healthy controls and patients with glaucoma [52, 53]. However, wearables that can track the VF while doing our daily activities are still in early stages.

One interesting device is AR DSpecs by Sayed et al., which are spectacles with integrated AR technology [32]. While calibrating, the glasses identify the size and location of scotomas and subsequently manipulate the view to fit video images of the unseen field into the remaining VF. It also carries an eye tracking system that can monitor gaze and fixation. AR DSpecs could possibly help monitor field loss by tracking changes in the VF, gaze, head movements and, if combined with GPS location, the walking pace. Another strategy described by Gestefelt et al. is a monocular eye movement tracker that predicts and monitors VF defects while patients watch TV or a movie; they used Eyelink 1000 from SR Research, however potential wearable options are described herein [54].

Another device that could possibly be used to track VF changes in the future is the innovative Apple Vision Pro (Apple Inc., Cupertino, CA), a see-through headset which combines VR and AR, with numerous applications under study [55, 56]. At present, the amount of user-generated data (such as eye tracking data) which can be exported from this device is limited.

Peripheral VF and central scotoma assessment are currently gold-standard outcome measures in various clinical trials for IRD and Age-Related Macular Degeneration (ARMD) [57]. Visual field assessment has many limitations including variability and poor compliance/reliability in children, in patients with nystagmus, and often in those with cognitive disabilities. Home monitoring ‘gamified’ vision tests are also an engaging alternative for young people [58].

## Visual acuity and central vision

Central vision issues are arguably more debilitating than those affecting the peripheral vision. Central visual loss is very common, given it affects people with ARMD and less common diseases such as inherited maculopathies and central serous chorioretinopathy, among others. Although multiple devices currently exist for home-monitoring purposes, wearable devices that are able to capture central vision features in the background are in early stages of development [14].

Wearable electronic vision enhancement systems have been available for nearly 30 years [59]. Although not widely adopted by people with vision impairment, in part due to their weight, cosmetic appearance and image lag [60, 61], it is possible that future systems may be more acceptable. On-device recording of the magnification needed to perform certain tasks could, in theory, be used as a marker of disease progression.

Zaman et al. created a prototype of a head-mounted display with VR/AR and mixed reality that is able to identify, characterise, and monitor a scotomatous monocular region, and modify it through binocular suppression [62]. Although only tested in healthy individuals with simulated scotomata, there are plans to test this device in patients with macular diseases [33].

AI-powered print-to-speech apps integrating video and audio processing such as Seeing AI (Microsoft; Redmond, WA, USA), Google Lookout (CA, USA), or Sullivan + (Tuat Corp, Daegu, Korea) are currently used for face recognition, identifying currency, colours, and reading [63]. These apps certainly have the capability to collect how often the individuals open the app, for what purpose and for how long, providing useful real-world central vision monitoring. Similarly, the AI-powered spectacle-mounted print-to-speech device Orcam MyEye (Jerusalem, Israel) could potentially collect usage parameters, and easily store it in the cloud with its internet connectivity [34].

## Functional vision: mobility and navigation

Mobility is frequently challenging for patients with visual impairment, and it is significantly associated with quality of life [64]. Analysing the physical ability to move efficiently and safely in an environment is a way of assessing functional vision, or how vision is used in everyday activities [57]. Characterising how a person interacts with the environment has become fundamental in understanding retinal conditions, being also pivotal to test if an intervention improves patients’ lives in a meaningful way [65]. To understand functional vision, many parameters need to be considered (including lighting conditions, obstacles, turns, contrast), in order to mimic real-life conditions as much as possible. Both multi-luminance mobility tests and VR settings have been developed to objectively measure how individuals with IRD perform under different lighting conditions [57, 66]. However, current electronic navigation aids combined with other wearables, AI, and IoT may serve as novel tools to assess functional vision in real-life rather than artificial settings.

Classic navigation aids are canes and guide dogs. Novel electronic aids including gyroscope, attitude and proximity sensors, cameras, and GPS tracking have recently been developed, providing patients with acoustic or haptic signals for improved obstacle detection and navigation (systematically reviewed elsewhere) [67, 68]. The Wearable Virtual Cane Network loses the cane altogether and replaces it with four sensors, on the waist, wrists and ankle, and a micro-vibration motor, becoming a hands-free aid [35]. This device also connects to Bluetooth, potentially acquiring whole-body navigation details and speed.

Different devices connect to smartphones for real-time mapping of the environment, GPS tracking, audio navigation, and to contact the patient’s network in case of emergencies [69–71]. A subset of these have been tested indoors too, to assist patients with daily activities in their own homes [71, 72]. Other technology (U-HAR, UCA-EHAR and JINS MEME) focuses on human activity recognition (HAR) and can incorporate data from the user’s head and eye movements with smart glasses, which gets analysed with a convolutional network, recognising and monitoring 7 to 10 different activities (talking, reading, dressing up, watching videos/TV, cooking, typing on a keyboard, riding a bicycle or walking) [36–38].

Strategies particularly useful for patients with RP include spectacles and a spectacle-mounted device (LV-3 and a see-through display device by Ikeda et al.) incorporating a display where the visual field is minified to fit on the patients’ narrower field, with enhanced contrast and brightness, facilitating vision for those with constricted field and nyctalopia, such as patients with RP [39, 40]. Lastly, Brodie et al. developed a wearable wireless head-positioning sensor for patients who underwent vitrectomy with intraocular gas, which provides real-time monitoring and audiovisual feedback on the accuracy of head positioning, increasing compliance in half of the volunteers who used it [41].

The devices above may provide a new generation of real-world continuous mobility monitoring, which could translate into improved outcome measures with less infrastructure needed and easier incorporation in worldwide multicentre studies.

## Conclusions and future directions / considerations

The digital health revolution prompts us all to engage with this change in paradigm and get acquainted with related technologies such as AI, AR and IoT. Wearable devices in Ophthalmology and in particular for retinal conditions have wide opportunities to detect risk factors (e.g., myopia), diagnose and monitor diseases (e.g., ARMD, RP), work as visual aids, and treat conditions (e.g., amblyopia) [73]. They are potentially able to simultaneously capture multiple aspects of the visual experience as we go about our daily activities, increasing our understanding of how people interact with their vision. Furthermore, they could create new clinical trial endpoints; for example, determining if duration of activities and distances/locations travelled differ after intervention, measuring a person’s stress levels while crossing a street, their heart rate when having visual acuity testing, or the light levels at which a patient prefers to work or read at home.

Many of the devices above show promise in generating relevant objective data. Possibly, soon there will be a wider use of wearables such as the Visual Behaviour Monitor or JINS MEME ES, which will collect both visual experience and environment parameters, VR/AR and mixed reality head-mounted displays monitoring our visual field as we do our daily activities, and functional vision sensors like the Wearable Virtual Cane Network, assessing the surrounding areas and characterising navigation. This will usher a new era of visual function information.

Clearly, there are still many challenges remaining including: (i) potential selection bias, with individuals having technical, financial or cultural barriers towards the use of portable intelligent devices; (ii) anonymisation and confidentiality of health information; (iii) storage and analysis of large amounts of data; (iv) technology and sensors development, allowing accurate data collection, improved interaction with technology, and integration with current healthcare records, and (v) appropriate standardisation against gold standard devices, proving that portable devices produce reliable data, with acceptable repeatability and variability in a large relevant population [12, 49, 74]. Home-monitoring devices, VR/AR devices, AI software, and wearables are in constant development and quality improvement, and still require exhaustive refinement alongside healthcare professionals and patients to become useful instruments that we can rely on. Some of the current issues faced are not detecting mild signs of disease in fundus imaging; having difficulty assessing retinal images with suboptimal visibility [75]; self- imaging OCT having a different scanning pattern than hospital-based OCT, leading to differences in retinal thickness measurements [76]; glasses and pupillary distance affecting the image projected on the retina by VR devices [77], among others. There are four domains to consider when evaluating digital health innovations: technical, clinical, usability, and cost-effectiveness [1]. Technical refers to the hardware and software development that results in fast and accurate data collection, with suitable cloud storage and encryption. Clinical refers to acquiring relevant health parameters, which may have an impact on the individual’s health. Usability considers the weight, comfort and aesthetics of the device, and lastly cost-effectiveness implies having an acceptable price, proving to be more affordable than traditional health consultations. Usability considerations have, for example, limited the uptake of wearable devices for vision enhancement in those with vision impairment.

The limitations of this review are the literature review in a non-systematic approach, possibly causing the omission of some scientific papers. The focus was ergonomic and small devices which could enable continuous monitoring. Devices such as not-see-through headsets were not considered as wearables given their weight and cosmetic impact, likely making them difficult for constant wearing and continuous data collection [20].

Much like the shrinking footprint of physical office spaces after the COVID-19 pandemic, the healthcare landscape is facing significant transformation. Telemedicine, home monitoring systems, wearable devices, and AI-driven retinal image diagnosis represent a new era in health management where a fundamental shift towards leveraging real-world data appears imminent. Furthermore, with the adoption of widespread, data acquisition methods, the need for costly traditional randomised clinical trials may diminish. This paradigm shift holds promise for enhancing patient engagement with research endeavours, while curbing the issue of patients lost to follow-up. Clinicians have a key role of collaborating with the development and fine-tuning of digital health innovations, as well as identifying opportunities where they can be leveraged to enhance our understanding of retinal diseases and improve patient engagement and outcomes.
